# Association between recurrence of acute kidney injury and mortality in intensive care unit patients with severe sepsis

**DOI:** 10.1186/s40560-017-0225-0

**Published:** 2017-05-22

**Authors:** Emilio Rodrigo, Borja Suberviola, Miguel Santibáñez, Lara Belmar, Álvaro Castellanos, Milagros Heras, Juan Carlos Rodríguez-Borregán, Angel Luis Martín de Francisco, Claudio Ronco

**Affiliations:** 1 Nephrology Service, IDIVAL-Hospital Marqués de Valdecilla, University of Cantabria, Santander, Spain; 2 Intensive Care Unit, IDIVAL-Hospital Marqués de Valdecilla, University of Cantabria, Santander, Spain; 3 School of Nursing, IDIVAL-Hospital Marqués de Valdecilla, University of Cantabria, Santander, Spain; 4 Department of Nephrology Dialysis and Transplantation, International Renal Research Institute of Vicenza (IRRIV), San Bortolo Hospital, Vicenza, Italy

**Keywords:** Acute kidney injury, Mortality, Recurrence, Sepsis

## Abstract

**Background:**

Acute kidney injury (AKI) occurs in more than half critically ill patients admitted in intensive care units (ICU) and increases the mortality risk. The main cause of AKI in ICU is sepsis. AKI severity and other related variables such as recurrence of AKI episodes may influence mortality risk. While AKI recurrence after hospital discharge has been recently related to an increased risk of mortality, little is known about the rate and consequences of AKI recurrence during the ICU stay. Our hypothesis is that AKI recurrence during ICU stay in septic patients may be associated to a higher mortality risk.

**Methods:**

We prospectively enrolled all (405) adult patients admitted to the ICU of our hospital with the diagnosis of severe sepsis/septic shock for a period of 30 months. Serum creatinine was measured daily. ‘In-ICU AKI recurrence’ was defined as a new spontaneous rise of ≥0.3 mg/dl within 48 h from the lowest serum creatinine after the previous AKI episode.

**Results:**

Excluding 5 patients who suffered the AKI after the initial admission to ICU, 331 patients out of the 400 patients (82.8%) developed at least one AKI while they remained in the ICU. Among them, 79 (19.8%) developed ≥2 AKI episodes.

Excluding 69 patients without AKI, in-hospital (adjusted HR = 2.48, 95% CI 1.47–4.19), 90-day (adjusted HR = 2.54, 95% CI 1.55–4.16) and end of follow-up (adjusted HR = 1.97, 95% CI 1.36–2.84) mortality rates were significantly higher in patients with recurrent AKI, independently of sex, age, mechanical ventilation necessity, APACHE score, baseline estimated glomerular filtration rate, complete recovery and KDIGO stage.

**Conclusions:**

AKI recurred in about 20% of ICU patients after a first episode of sepsis-related AKI. This recurrence increases the mortality rate independently of sepsis severity and of the KDIGO stage of the initial AKI episode. ICU physicians must be aware of the risks related to AKI recurrence while multiple episodes of AKI should be highlighted in electronic medical records and included in the variables of clinical risk scores.

## Background

Acute kidney injury (AKI) occurs in more than half critically ill patients admitted in intensive care units (ICU), being sepsis and septic shock the main causes of AKI in ICU patients [1, 2]. In the recently reported multinational AKI-EPI study, 57.3% of 1802 patients in ICU developed AKI [1]. Patients affected by AKI present higher risk of mortality, further chronic kidney disease (CKD) and end-stage renal disease (ESRD) [1, 3–5] and have a major impact on healthcare resources [6].

The important advance in AKI severity grading achieved by recent definitions and classification systems such as RIFLE, AKIN, KDIGO and creatinine kinetics has allowed identifying a specific metrics in epidemiology and outcome studies [7–10]. Furthermore, these four classifications have demonstrated the relationship between AKI severity and patient outcomes (mortality and hospital length of stay) and have improved our knowledge about AKI epidemiology [1, 11–15]. In these circumstances, the identification of all the AKI-related variables is quintessential to predict AKI occurrence, severity and outcome.

In fact, AKI severity is not the only factor influencing middle and long-term outcomes. Both AKI duration and recurrence could influence morbidity, mortality and healthcare cost associated to AKI. On the one hand, several authors have suggested that the duration of AKI is as important as severity with regard to outcomes [16–18]. On the other hand, some studies have demonstrated that AKI recurrence after hospital discharge can take place up to 30% after the initial AKI-related hospital admission and is associated to a higher risk of mortality and CKD [19, 20]. Although, currently no uniform definitions of AKI recovery and recurrence exist, there is a growing interest in increasing the knowledge about trajectories of recovery after an AKI episode [21, 22]. In this sense, a consensus conference was held in San Diego in 2015 focusing on ‘Persistent AKI and renal recovery’ [22]. Specifically, little is known about the rate and consequences of AKI recurrence during ICU and hospital stay. Our hypothesis was that AKI recurrence in septic patients during ICU stay is independently associated with mortality. To contrast this hypothesis, our first aim was to determine whether in-ICU AKI recurrence is an independent factor associated with mortality in comparison with patients without AKI and with patients who only suffered one AKI episode, showing a dose-response pattern. The second aim was to address the importance of AKI recurrence on the risk of mortality with independence of severity (KDIGO stage) of the first AKI. Last, our third objective was to determine the association between AKI recurrence and mortality in patients who recovered completely from their first AKI.

## Methods

A prospective observational cohort study was carried out in all patients (over 17 years of age) admitted to the ICU—of ‘Marqués de Valdecilla’ University Hospital in Santander (Spain)—with severe sepsis/septic shock according to the definitions proposed by the SCCM/ESICM/ACCP/ATS/SIS consensus conference (i.e. the presence of arterial hypotension and/or persistent signs of tissue hypoperfusion refractory to the intravenous administration of fluids [20 ml/kg], and requiring the infusion of vasoactive drugs) [23]. Enrollment occurred from April 2008 to September 2010. Patients with chronic kidney disease under renal replacement therapy or who had received a kidney transplant were excluded.

Clinical and demographic characteristics of all patients, including age, gender, previous diagnosis of hypertension, diabetes mellitus, chronic obstructive pulmonary disease (COPD), chronic heart failure (CHF) or cancer, immunosuppressive state (AIDS, neutropenia [neutrophil count <1 × 109/L], exposure to glucocorticoids [>0.5 mg/kg for >30 days] and/or immunosuppressive or cytotoxic medications, solid organ transplantation, allogeneic or autologous stem cell transplantation, haematological malignancy or solid tumour), the source of infection, as well as Acute Physiology and Chronic Health Evaluation II score and Sequential Organ Failure Assessment score at ICU admission, mechanical ventilation necessity, use of vasopressors and length of ICU and hospital stay were recorded. Leukocyte number, lactate, C-reactive protein and procalcitonin values were collected at ICU admission. Serum creatinine was measured daily while the patients were in ICU. Baseline serum creatinine was defined by the most recent available value between 7 and 365 days before hospital admission. Baseline glomerular filtration rate (GFR) was estimated by 4-variable modification of diet in renal disease (MDRD) equation [24]. In 16 (4%) patients with no available baseline creatinine, it was calculated from the simplified MDRD formula assuming a GFR of 75 ml/min per 1.73 m^2^ as recommended by the acute dialysis quality initiative (ADQI) workgroup [7]. We defined and staged AKI according to KDIGO serum creatinine criteria [9]. In-ICU recurrent AKI was defined as a new spontaneous rise of ≥0.3 mg/dl within 48 h from the lowest serum creatinine after the previous AKI episode, with partial or full recovery. We defined complete recovery when the patient serum creatinine returned to baseline creatinine or below. Partial recovery was defined when the patient was off renal replacement therapy and serum creatinine started to decrease after the peak value but failed to return to baseline creatinine. Rises of serum creatinine after renal replacement therapy withdrawal were not defined as AKI recurrences. In-hospital and at 90-day mortality were prospectively collected, and mortality at the end of follow-up was retrospectively collected in 2014, being analysed as dependent variables.

Categorical variables were expressed as percentages and continuous variables as median and interquartile rank (IQR). Statistical differences between groups were analysed by chi-square test or Fisher exact test when appropriate for categorical variables, and the non-parametric Mann-Whitney *U* test was used for continuous variables.

We tested the equality of survival distributions for no AKI, one AKI existence and AKI recurrence by using the log-rank (Mantel-Cox) test and Kaplan-Meier survival curves. As KDIGO stage of the first AKI independently related to mortality, we additionally stratified by KDIGO stage after excluding patients without AKI, testing the equality of survival distributions for one AKI and AKI recurrence in patients with KDIGO 2 and 3 stages separately.

We estimated hazard ratios (HRs) to measure associations. We estimated adjusted HRs and their corresponding 95% confidence intervals (95%CI) using proportional hazards Cox regression models. We adjusted for the following variables: gender, age, mechanical ventilation necessity, APACHE score and baseline estimated GFR (ml/min/1.73 m^2^). When patients without AKI were excluded from the analysis, KDIGO stage and ‘complete recovery’ were also included in the multivariable models. The alpha error was set at 0.05, and all *p* values were two sided. We conducted all statistical analyses using IBM SPSS Statistics version 22.0.

## Results

**Fig. 1:**
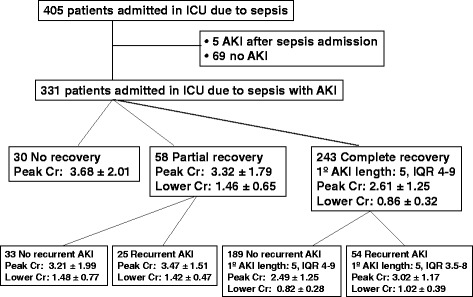
Flow chart of the study population. Abbreviations: *ICU* intensive care unit, *AKI* acute kidney injury, *Cr* serum creatinine, *IQR* interquartile range

Main characteristics of the 405 patients included in the cohort are shown in Table 1. Mean follow-up was 956 (IQR 28–1662) days. Throughout the study, they were no losses of follow-up during hospital stay. In 17 patients (4.25%), it was not possible a complete ‘follow-up’ up to 90 days after hospital discharge. In 25 patients, the follow-up was <1 year, and in 33 patients, the follow-up was <2 years. Five patients suffered AKI after the initial admission in ICU due to sepsis, and they were excluded from the analysis. Three hundred thirty-one patients out of the 400 patients (82.8%) developed at least one in-ICU acute kidney injury (AKI) according to KDIGO classification. Among them, 72, 6 and 1 patients suffered 2, 3 and 4 AKI episodes, respectively, so 79 out of 400 patients (19.8%) developed recurrent AKI episodes during their ICU stay. The flow chart of study population is shown in Fig. 1.

**Table 1:** Baseline characteristic in all patients, and in relation to AKI recurrence risk

	All patients	No AKI	Patients with in-ICU AKI		
			AKI = 1	AKI ≥2 (recurrent)	
	*N* = 405	*N* = 69	*N* = 252	*N* = 79	*p* value
Age (years), median [IQR]	68.21 [56.52–77.70]	55.84 [49.02–69.52]	69.28 [58.07–78.23]	74.29 [64.72–79.16]	*0.027*
Gender (male)	68.9%	56.5%	71.0%	72.2%	*0.848*
Hypertension	47.7%	27.5%	50.8%	58.2%	*0.248*
Diabetes mellitus	18.3%	4.3%	22.6%	17.9%	*0.380*
COPD	14.8%	13.0%	14.3%	19.0%	*0.313*
CHF	6.4%	4.3%	7.1%	6.3%	*0.804*
Cancer	13.6%	10.1%	13.9%	15.2%	*0.773*
Immunosuppressive state	17.8%	11.6%	19.4%	17.7%	*0.734*
Source of infection					*0.122*
Intra-abdominal	30.9%	29.0%	31.0%	32.9%	
Lung	37.3%	52.2%	33.3%	35.4%	
Endocarditis	0.5%	0.0%	0.0%	1.3%	
Line related	1.5%	1.4%	2.0%	0.0%	
Urinary tract	12.3%	8.7%	15.5%	6.3%	
Skin and soft infection	2.0%	0.0%	2.0%	3.8%	
Unknown/others	15.6%	8.7%	16.3%%	20.3%	
Leukocytes, median [IQR]	13.60 [6.90–21.00]	12.6 [5.15–23.55]	13.60 [7.00–20.45]	15.50 [7.35–21.58]	*0.441*
Lactate (mg/dl), median [IQR]	23.0 [15.0–37.3]	[–]	26.0 [16.0–41.0]	26.0 [17.0–47.0]	*0.437*
Vasopresors	84.3%	83.8%	85.6%	79.7%	*0.214*
Septic shock	85.4%	85.5%	86.4%	82.3%	*0.366*
APACHE, median [IQR]	20 [16–25]	16 [12–19]	20 [16–27]	23 [19–27]	*0.032*
SOFA, median [IQR]	8 [6–10]	7 [5–8]	9 [7–11]	9 [6–11]	*0.952*
Mechanical ventilation	51.1%	54.4%	49.4%	51.9%	*0.699*
C-reactive protein (mg/l), median [IQR]	19.4 [10.30–27.50]	18.6 [7.85–25.45]	20.4 [10.7–29.20]	18.3 [11.05–27.55]	*0.673*
Procalcitonin (ng/l), median [IQR]	10.24 [2.54–30.37]	4.11 [1.25–10.91]	13.11 [3.31–34.00]	13.29 [2.32–41.90]	*0.749*
Baseline creatinine (mg/dl), median [IQR]	0.97 [0.80–1.13]	0.88 [0.72–1.00]	0.95 [0.80–1.15]	1.05 [0.90–1.20]	*0.022*
Baseline estimated GFR (ml/min/1.73 m^2^), median [IQR]	72.50 [58.36–87.86]	81.91 [64.79–102.55]	72.74 [57.23–86.47]	66.18 [53.84–77.55]	*0.030*
ICU stay (days), median [IQR]	4.99 [2.99–11.47]	4.99 [1.99–8.48]	3.98 [2.99–10.97]	9.97 [4.99–17.95]	*<0.001*
Hospital stay (days), median [IQR]	16.95 [10.22–30.92]	15.96 [10.97–32.41]	15.96 [9.97–28.92]	19.94 [13.96–34.90]	*0.003*
KDIGO AKI stages					*0.054*
1, *n* (%)	100 (24.7%)	–	79 (31.3%)	21 (26.6%)	
2, *n* (%)	121 (29.9%)	–	98 (38.9%)	23 (29.1%)	
3, *n* (%)	110 (27.2)	–	75 (29.8%)	35 (44.3%)	
Maximal creatinine (mg/dl), median [IQR]	2.10 [1.40–3.16]	1.00 [0.84–1.10]	2.30 [1.70–3.19]	2.99 [2.10–4.00]	*<0.001*
Intra-hospital mortality, *n* (%)	104 (25.7%)	7 (10.1%)	60 (23.8%)	35 (44.3%)	*<0.001*
90-day mortality, *n* (%)	112 (27.7%)	8 (11.6%)	64 (25.4%)	38 (48.1%)	<0.001
End of follow-up mortality, *n* (%)	190 (46.9%)	22 (31.9%)	113 (44.8%)	53 (67.1%)	*0.001*

Variables related to AKI recurrence are also shown in Table 1. Patients with AKI recurrence were significantly older and with lower baseline estimated GFR. APACHE score was also higher. Crude mortality was 102/400 (25.5%) for in-hospital mortality, 110/400 (27.5%) at 90 days of follow-up and 188/400 (47%) at the end of follow-up. Ninety-day survival was 59.3, 44.0, 86.7 and 55.3% for patients with partial recovery without AKI recurrence, partial recovery with recurrence, complete recovery without recurrence and complete recovery with recurrence, respectively.

**Fig. 2:**
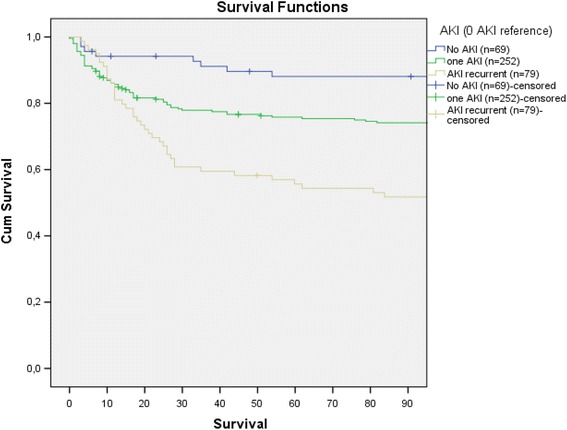
90-day survival curves including patients without AKI

In relation to our first objective, statistically significant different survival distributions were observed when we ordinal categorised ‘AKI existence’ into ‘patients without any AKI episode’, ‘only one AKI’ and ‘two or more AKI episodes (in-ICU recurrent AKI) (log rank *p* < 0.001) (Fig. 2, Table 1). By Cox regression analysis, significant dose-response patterns (adjusted *p* trends ≤0.021) were also found. The greater the number of AKIs, the greater the association for ‘intra-hospital’, ‘90 days’ and ‘end of follow-up’ mortality, with independence of sex, age, mechanical ventilation necessity, APACHE score and baseline estimated GFR (Table 2).

**Table 2:** Hazard ratios for in-ICU AKI existence, in relation to mortality

	Vital status (intra-hospital)					Vital status (90-day)					Vital status (end of follow-up)				
	Survival (*N*)	Death (*N*)	HR	(95% CI)		Survival (*N*)	Death (*N*)	HR	(95% CI)		Survival (*N*)	Death (*N*)	HR	(95% CI)	
Including patients without AKI (*N* = 400)	298	102				290	110				212	188			
No AKI (*n* = 69)	62	7	1	–		61	8	1	–		47	22	1	–	
AKI = 1 (*n* = 252)	192	60	1.68^a^	0.74	3.82	188	64	1.54^a^	0.71	3.34	139	113	1.00^a^	0.61	1.63
AKI ≥2 (*n* = 79)	44	35	2.73^a^	1.15	6.51	41	38	2.57^a^	1.13	5.83	26	53	1.61^a^	0.93	2.77
*Linear p trend*			*0.006*					*0.005*					*0.021*		
Excluding patients without AKI (*N* = 331)	236	95				229	102				165	166			
AKI = 1 (*n* = 252)	192	60	1	–		188	64	1	–		139	113	1	–	
AKI ≥2 (*n* = 79)	44	35	2.48^b^	1.47	4.19	41	38	2.54^b^	1.55	4.16	26	53	1.97^b^	1.36	2.84

^a^HR = hazard ratio adjusted for sex, age, mechanical ventilation necessity, APACHE score, and baseline estimated glomerular filtration rate (GFR)

^b^HR = hazard ratio adjusted for sex, age, mechanical ventilation necessity, APACHE score, baseline estimated GFR, complete recovery and KDIGO stage

KDIGO stage of the first AKI, independently related to intra-hospital (adjusted HR per each increase of stage = 1.45, 95% CI 1.10–1.91), 90-day (adjusted HR per each stage increase of severity = 1.31, 95% CI 1.01–1.71) and end of follow-up mortality (adjusted HR per each increase = 1.28, 95% CI 1.05–1.57).

**Fig. 3:**
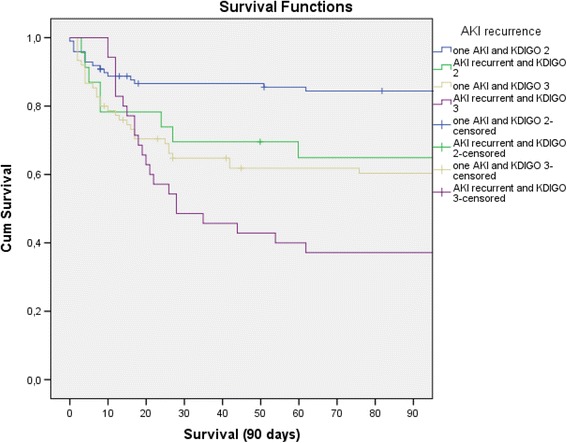
90-day survival curves as a function of KDIGO stages, excluding patients without AKI and restricting to patients with KDIGO 2 or 3 stages

Regarding our second objective, a specific analysis was conducted with exclusion of non AKI patients, in order to address the importance of AKI recurrence with independence also of severity of the first AKI. Excluding 69 patients without AKI, in-hospital (adjusted HR 2.48, 95% CI 1.47–4.19), 90-day (adjusted HR 2.54, 95% CI 1.55–4.16) and end of follow-up (adjusted HR 1.97, 95% CI 1.36–2.84) mortality rates were significantly higher in patients with recurrent AKI, independently of the covariates above and KDIGO stage and ‘complete recovery’ (Table 2). Restricting to patients with KDIGO 2 or 3 stages in the first AKI, survival curves were also significantly lower for AKI recurrent patients in each KDIGO stage (log rank *p* < 0.001) (Fig. 3).

In relation to our third objective, 243 patients out of the 331 patients admitted in ICU due to sepsis with AKI recovered completely from their initial AKI episode. Analysing only this group of patients with complete recovery (*N* = 243), the recurrence of AKI remained as an independent risk factor for further mortality at 90 days (HR 3.21, 95% CI 1.74–5.92, *p* < 0.001) and at the end of follow-up (data not shown in tables).

## Discussion

The main finding of our study is that the development of a new episode of AKI during ICU stay after the first AKI episode related to sepsis is associated with a higher mortality rate and increases the mortality rate. The mortality risk rises more than double if the patient suffers from two or more AKI episodes during the same admission. Similarly, Siew et al. reported that patients admitted to hospital with recurrent AKI—within 12 months of discharge from the previous hospitalization with AKI—near doubled the death rate [19]. It is known that diabetic patients with AKI recurrence after previous hospital discharge are at risk for stage 4 CKD. However, we did not analyse residual renal function in our study [20]. Obviously, in our cohort, this increment in mortality comes up after the first 5–10 days (Fig. 2) because AKI recurrence takes some time to develop. As previously reported [15], KDIGO stages of the first AKI episode are independently related to mortality. Remarkably, using KDIGO serum creatinine criteria to define AKI, we found that such small elevations of creatinine as 0.3 mg/dl relate to a higher mortality risk when they appear after a first AKI event and this increment in mortality was independent of first episode KDIGO classification and severity of sepsis estimated by APACHE score [9].

Moreover, we found that in-ICU AKI recurrence was frequent, taking place in up to 20% of patients admitted due to sepsis. It is expected that the rate of recurrence after AKI related to different aetiologies should be lower because sepsis is the most frequent AKI cause [1]. AKI recurrence rate after hospital discharge is experienced by 25–30% patients, although in-hospital AKI recurrence rate has not been previously reported [19, 20]. In addition, some patients suffered a third and a fourth in-ICU AKI episode, which, in time, can even increase more the risk of death, although we could not confirm this case because only 7 patients developed so numerous AKI episodes.

As pointed out by Siew et al., in order to prevent further AKI recurrence, we need to identify patients at highest recurrence risk [19]. Age and baseline estimated GFR clearly related to a higher risk of recurrence in our cohort study, and both are well-known risk factors for AKI development [25]. By contrast, we did not find any relationship between recurrence and gender or comorbid risk factors (Table 2). Siew et al. reported that age, baseline renal function and comorbid conditions such as congestive heart failure, advanced liver disease, dementia, diabetes and coronary artery disease were associated with recurrent AKI after discharge for initial hospital admission in 11,683 patients [19]. On the one hand, the lower number of patients included in our study can prevent us from detecting the influence of these risk factors. It is expected that older patients show more comorbid conditions that can place them at a higher risk for AKI recurrence. On the other hand, all our patients developed the first AKI episode due to sepsis and this can limit the influence of other comorbid risk factors.

In our study, severity of sepsis estimated by Sequential Organ Failure Assessment (SOFA), number of leukocytes, lactate, C-reactive protein, procalcitonin and use of vasopressors did not relate to AKI recurrence. Conversely, the severity of the first AKI episode was associated with a higher AKI recurrence as well as severity of sepsis as assessed by APACHE score. Although without reaching statistical significance, 44% patients with in-ICU recurrence had previously suffered a KDIGO stage 3 AKI episode, whereas only 30% patients without recurrence had shown a similar AKI stage. Maximal creatinine in the first AKI episode and baseline estimated GFR were also higher in patients with further recurrence. In this sense, older patients admitted in ICU due to sepsis, with higher APACHE scores and worse renal function and a more severe AKI episode can be identified as at a higher rate of AKI recurrence in our cohort.

The main advantage of our study was that we defined KDIGO stages and AKI recurrence analysing one-by-one daily creatinine for each patient. For example, a rise of creatinine ≥0.3 mg/dl after the initial AKI episode was only considered AKI recurrence when the previous AKI episode was recovering and not when it was due to continuous or intermittent renal replacement therapy withdrawal. AKI recurrence can be hard to record unless prospectively reported in the medical history of the patient or in electronic databases. Due to the association of AKI recurrence with mortality rate, we suggest that the ICU physicians must be aware to its risk and add this diagnosis in the clinical records and registers together with the AKI index episode.

Our study has several limitations. First, we performed a single-centre study and the number of patients included was not high enough to detect the influence of several risk factors previously related to AKI [19, 25]. Whereas one of the advantages of multicentre studies is the high number of patients that can be enrolled, studies carried out in single centres are more homogeneous with respect to inclusion criteria and type of care of the patients. In our case, all patients were included if they fulfilled severe sepsis/septic shock definition according to the SCCM/ESICM/ACCP/ATS/SIS consensus conference [23].

Second, we lost to follow up 4.25% of patients at 90 days because our ICU is part of a tertiary care hospital and some of these patients were discharged to different healthcare systems. We cannot rule out that this rate of follow-up losses had an impact on the reported results, but meaningfully, all the patients were followed up throughout the whole hospital admission until ‘death’ or ‘discharge home’ and there were no in-hospital losses. Third, we did not use urine output to define AKI. Although urine volume rate is part of the AKI definition [9], most AKI staging studies are based on serum creatinine levels alone and do not include urine output data [26]. The course of ICU stay in patients with sepsis should be closely monitored in order to detect recurrent episodes of AKI [27–29].

## Conclusions

To conclude, we performed a single-centre observational study and detected that AKI can recur in up to 20% of patients who suffer a sepsis-related AKI during the initial hospital admission episode. This recurrence is associated with a higher mortality rate independently of several covariates such as initial AKI and sepsis severity, and the association between AKI recurrence and mortality seems to be also present in patients who recovered completely from their first AKI. If these findings are confirmed in larger subsequent multicenter studies, it may be advisable for ICU physicians to be aware of AKI recurrence risk and to add the number of AKI episodes along with their severity and duration in clinical and electronic records to establish the global influence of AKI episodes on patient outcome.

## Abbreviations

95%CI: 95% confidence intervals
ACCP: American College of Chest Physicians
ADQI: Acute dialysis quality initiative
AIDS: Acquired immune deficiency syndrome
AKI: Acute kidney injury
AKIN: Acute kidney Injury network
APACHE: Acute physiology and chronic health evaluation
ATS: American thoracic society
CHF: Chronic heart failure
CKD: Chronic kidney disease
COPD: Chronic obstructive pulmonary disease
ESICM: European Society of Intensive Care Medicine
ESRD: End-stage renal disease
GFR: Glomerular filtration rate
HR: Hazard ratio
ICU: Intensive care units
IQR: Interquartile range
KDIGO: Kidney Disease/Improving Global Outcomes
MDRD: Modification of diet in renal disease
RIFLE: Risk, injury, failure, loss of kidney function, and end-stage kidney disease
SCCM: Society of Critical Care Medicine
SIS: Surgical Infection Society
SOFA: Sequential Organ Failure Assessment
